# Society Reports—Atlanta Medico-Chirurgical Association

**Published:** 1878-06-20

**Authors:** 


### SOCIETY REPORTS.

### Atlanta Medico-Chirurgical Association.

(Reported by Dr. Word.)

Monday Evening, May 20.

Dr. J. J. Knott in the chair.

Dr. A. R. Alley reported a case of jaundice in a lady twenty years of age. On first visit, found patient languid, with slow pulse, pain and tenderness in hypochondriac region, cold, clammy perspiration, etc. Next morning, the yellow tinge of jaundice appeared. The patient had been in trouble a few days previous to attack, which was probably the predisposing cause. Gall-stones passed. The treatment used in this case was a glass of cider with the yolk of an egg and a tablespoonful of brown sugar given every morning for about ten days, under which remedy the patient gradually recovered. The remedy is laxative and alterative, and has a tendency to impart tone to the stomach.

Dr. A. asked the views of members as to the nature of jaundice, its treatment, etc.

Dr. Knott said he thought it probable that jaundice was often caused by neuralgia of the stomach, producing contraction of the biliary ducts by impressing the solar plexus of nerves. He had a patient subject to gastrodynia, who invariably becomes jaundiced after each attack. He had used, with good results, in this disease, the following prescription :

℞.—Carbonate ammoniæ......grs. xc.

Lemon juice................oz. ij.

Dose—a teaspoonful every two to three hours. It effervesces and forms the citrate of ammonia. It allays nausea, is diuretic, and acts as a gentle aperient.

Dr. Word stated that jaundice resulted from obstruction to the free exit of bile from the liver or gall-bladder into the duodenum, the icteric hue being caused by the retention of the coloring matter of the bile in the blood. The obstruction might occur in many ways. Engorgement, or congestion of the liver interfering with free secretion and elimination, inspisated bile not flowing freely through the ducts, duodenitis, congestion or thickening of the mucous membrane of the duodenum, constipation or a loaded condition of the colon making pressure upon the liver, interfering with the portal circulation, or otherwise impeding the function of the liver; biliary calculi accumulating in the gall-bladder, or lodging in the gall-duct. Mental emotion, by impressing the nerves or unfavorably affecting the process of digestion, might produce the disease; but in the case of Dr. Alley, it would seem plain that the obstruction was mechanical—caused by gall-stones. The severe spasmodic pain, which was caused by the passage of gall-stones, very much resembles what is somewhat vaguely termed, in the books, gastralgia, gastrodynia, or neuralgia of the stomach.

Relief soon follows after a gall-stone has passed, though it may be sometime before the yellow tinge of the eye and skin disappears. If, however, the gall-stones accumulate and cannot be discharged, there will be repeated paroxysms of intolerable pain, attended with rigors, profuse sweats, hiccup, coma, and death.

Dr. Word thought the rational treatment of jaundice is, first, the administration of an emetic, followed by a bilious cathartic, as best calculated to remove obstructions from whatever cause, and prepare the system for the use of acids, diuretics, etc.

He mentioned a remedy which had recently been very highly extolled, in jaundice, said by the eclectics to be a specific—the *Chionanthus Virginica* (Grandsir Graybeard)—a species of ash which grows in our swamps. An infusion of the root is preferred. He had not tried this remedy.

Dr. Knott differed with Dr. Word. Mechanical causes of obstruction were difficult to detect, but thought that the passage of a gall-stone need not be mistaken for neuralgia. There is a well defined terderness from stone, and the pain is more circumscribed than in neuralgia. He mentioned a fatal case of jaundice in his experience, attended with excruciating pain in the right side, to relieve which, morphia, hypodermically, was used. Opium was found necessary to keep down pain. The jaundice was not general, but appeared in spots. Patient directed to keep under the influence of opium, as the recurrence of the excruciating attacks of pain might cause rupture of the gall-bladder, which, he believes, did finally occur, the friends of the patient having, contrary to his advice, left off the opiates, as a result of which the paroxysm of pain returned, tympanetic distension and tenderness of abdomen followed, indicating rupture, and peritonitis, from which the patient died.

MONDAY EVENING, June 3.

Dr. J. J. Knott reported a case of paralysis of lower extremities, in a child two years old, from reflex action, caused by extreme thickening of the mucous membrane of the prepuce. He did not believe that adhesion of the prepuce to the glans was necessarily the cause of the paralysis in such cases. In this case, the irritation seemed to proceed from the mucous membrane of the prepuce, which was not adherent to the glans, and admitted of a considerable degree of retraction. The case was relieved by circumcision.

Dr. Olmstead thought that the condition of this case, as described by Dr. Knott, was sufficient to account for the paralysis, as cases occurred in the female from simple enlargement of the clitoris.

Dr. H. B. Lee reported a case of strangury in a negro man forty years of age. The meatus was very much contracted, and there was great tenderness over the lumbar region, and great nervous sensibility. The case was relieved by an operation for stricture of the meatus.

Dr. J. C. Olmstead, having seen the case with Dr. Lee, confirmed the facts as mentioned, and stated that the meatus was contracted to a remarkable small size.

Dr. J. J. Knott remarked that authors had said very little on this subject, and but little was known, except what was gathered from specialists. He thought that elongation of the prepuce was, in many cases, the cause of this condition. Had treated a case in a young man who had to be first circumcised, and then the meatus enlarged by incision.

Dr. Olmstead stated that a monogram upon the subject of anterior contraction of the meatus had been published by Dr. Otis, of New York.

